# Image-based dataset of artifact surfaces fabricated by additive manufacturing with applications in machine learning

**DOI:** 10.1016/j.dib.2022.107852

**Published:** 2022-01-21

**Authors:** Jiaqi Lyu, Javid Akhavan, Souran Manoochehri

**Affiliations:** Stevens Institute of Technology, 1 Castle Point Terrace, Hoboken, NJ 07030, USA

**Keywords:** 3D printing, Anomaly detection, Point cloud, Process monitoring, Shallow and deep learning, Laser surface profiling, Smart manufacturing, FFF machine optimization

## Abstract

Fused Deposition Modeling (FDM), also known as Fused Filament Fabrication (FFF), is the most widely used type of Additive Manufacturing (AM) technology at the consumer level. This technology severely suffers from a lack of online quality assessment and process adjustment. To fill up this gap, a high-speed 2D Laser Profiler KEYENCE LJ-V7000 series is equipped above an FDM machine and performs a scan after each print layer. The point cloud of the upper surface will be processed and transformed into a 2D depth map to analyze the in-plane anomalies during the FDM fabrication process. The author used the above data to categorize the surface quality into four categories: under printing, over printing, normal, and empty regions. The author showed the effectiveness of data in detecting print anomalies, and further work can be done to show the application of more advanced algorithms towards a better detection accuracy or to present a novel way to approach the data and detect a broader range of anomalies.

**Specification Table**

**Table:** 

Subject	Engineering, Computer Science
Specific subject area	Mechanical Engineering, Manufacturing Engineering, Image processing, Image identification, Image classification, computer vision
Type of data	Image
How the data were acquired	A Laser Surface Profilometer acquired point clouds by the KEYENCE LJ-V7000 series. Experts labeled a portion of them for anomalies.
Data format	Raw and processed
Description of data collection	A 2D Laser Profiler KEYENCE LJ-V7000 series is equipped above an FDM machine. After each layer deposition, the laser scanner acquires the 3D point cloud of the upper surface. Then the 3D point cloud is pre-processed to achieve the target surface of the part. Then the top surface is segmented from the point cloud and converted to a 2D depth image provided with this article.
Data source location	• Institution: Stevens Institute of Technology • City/Town/Region: Hoboken NJ • Country: USA
Data accessibility	Repository name: Mendeley data Data identification number: 10.17632/zyz6cznm5h.3 Direct URL to data: https://data.mendeley.com/datasets/zyz6cznm5h/3
Related research article	For an article that has been accepted and is in press: J. Lyu, J.A.T. Boroujeni, S. Manoochehri, In-situ laser-based process monitoring and in-plane surface anomaly identification for additive manufacturing using point cloud and machine learning, Proceedings of Int. Des. Eng. Tech. Conf. Comput. Inf. Eng. Conf. Vol. 85376. American Society of Mechanical Engineers, 2021. [1]

**Value of the Data**

• *The dataset provided contains several parts’ top surface information which can be used to study many aspects of the print.*
• *The dataset provided and the techniques used can be further integrated with many 3D printing machines to study the undergoing print.*
• *This dataset is expected to benefit scientists working on process monitoring and advancement, also developers of online control techniques for AM machines.*
• *The dataset provided can be used by researchers to develop more capable networks with more comprehensive detection capacities*
• *The dataset can be used to compare new algorithms’ efficiency and prediction accuracies.*

## 1 Data Description

The dataset presented in this article consists of 434 scan files from the top surface of the artifacts under study. Each scan file is pre-analyzed and threshold to generate color map data to represent the surface quality better. All the scan files are summarized and stored in a struct file with branches as follows:

1) Heightmap image of size [300 × 300 × 3 uint8]
2) Labels [10 × 10 double]
3) Part's name or number as a string

**Fig. 1:**
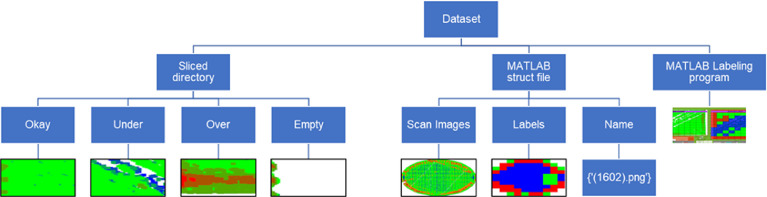
Structure of the dataset.

Each height map data is divided into a 10by10 grid (100 segments in total), and professionals labeled each segment into four categories: (a) Over Printing Situation, (b) Normally Printed Situation, (c) Under Printing Situation, (d) Empty. After acquiring labels, each image was cropped into 100 smaller images and then stored in a separate folder based on the label. Table 1 summarizes the amount of data available for each category and the proportion used for training, validation, and testing. Four directories containing all the segments are also attached to the dataset. On top of the data, we have also attached a MATLAB-based User Interface (U.I.) developed for labeling this dataset. Fig. 1 shows the structure of the dataset.

**Table 1:** Number of training, validating, and testing images before data augmentation.

CATEGORY	NAME	TRAINING	VAL	TEST	TOTAL
1	Over	8,587	2,454	1,227	12,268
2	Okay	9,259	2,646	1,323	13,228
3	Under	10,308	2,945	1,473	14,726
4	Empty	2,225	636	318	3,179
TOTAL		30,379	8,681	4,341	43,401

## 2 Experimental Design, Materials and Methods

The dataset presented in this study is captured by an online laser-based process monitoring and anomaly identification system for the FDM machine. This system equips with a non-contact laser scanner to obtain the top surface profile of each layer during the FDM fabrication process. This section describes the experimental setup, the pre-processing steps for the dataset, and the data labeling steps.

## 3 Experimental Setup

**Fig. 2:**
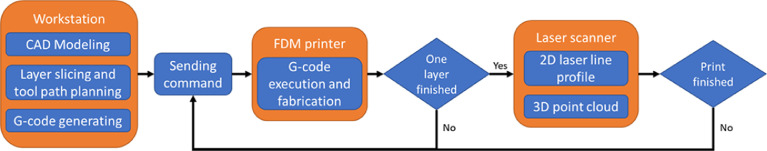
Schematic of the online laser-based process monitoring system.

**Fig. 3:**
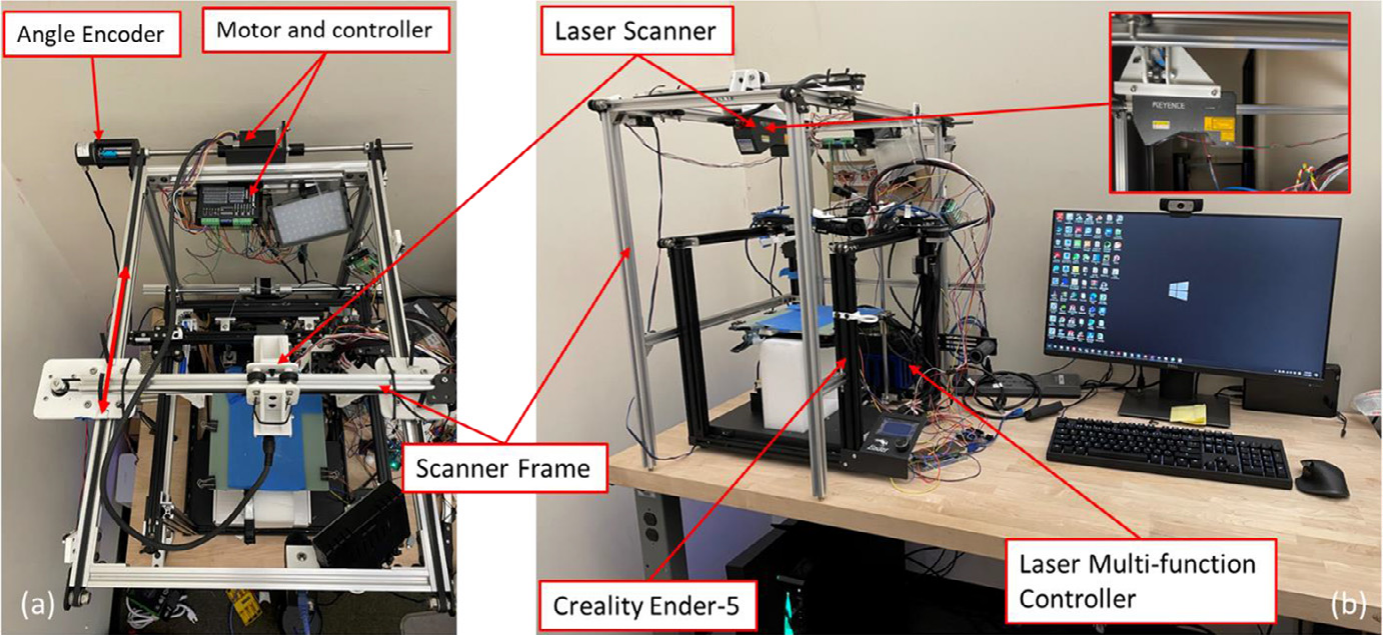
Experimental setup (a) the top view (b) all the components of the 3D printer-scanner system.

In this study, the FDM machine used is Creality ender 5 with 220mm × 220mm × 300mm building volume. The schematic of the online laser-based process monitoring system is shown in Fig. 2. A high-speed 2D Laser Profiler KEYENCE LJ-V7000 series is equipped above the FDM machine. The laser scanner includes a laser line emitter and built-in camera packaged with a fixed relative position. The scanner is calibrated from the factory and has a 1 µm resolution in the Z direction. The measurement range of the laser scanner is ±48 mm from the reference position, which is 200 mm below the bottom of the sensor. An artifact is designed in SOLIDWORKS 3D CAD design software for each print and converted into Standard Tessellation Language (STL) with American Standard Code for Information Interchange (ASCII) codes. The Simplify3D Software is used to slice the STL model and generates the G-code of the artifact. Then the FDM machine starts printing after receiving the G-code from the workstation. After each layer is completed, the scanner frame motion mechanism will drive the laser scanner to move above the fabricated layer with constant speed. At the same time, an angle encoder continuously triggers the scanner to perform measurements. The limit switches are there to make sure the scan is limited to the platform area of the FDM machine. Then the 3D point cloud of the upper surface is acquired from the laser scanner. The experimental setup is shown in Fig. 3.

### 3.1 Point cloud processing

The 3D point cloud acquired from the laser scanner includes noise that needs to be removed before further analysis. The mechanical vibration commonly causes noise during the scanning process and the shadow effect characteristics of the optical scanning sensor. This study uses three steps to pre-process the 3D point cloud, including replacing missing measurement, removing measurement noise, and transformation.

### 3.2 Step 1: replace missing measurement values

When the target is out of detecting range during the laser scanning process, or does not capture enough information, the laser scanner generates missing measurements values, flagged as Nan in this laser scanner. The raw data obtained from the laser scanner is a 2D depth matrix in the form of m×n. To replace the missing measurement values, the median operator with a 3×3 window is utilized [2]. If missing values surround the missing value, the pixel will be removed. Then filled 2D depth dataset is converted to point cloud form represented as (x,y,z) spatial coordinates.

### 3.3 Step 2: remove measurement noise

**Fig. 4:**
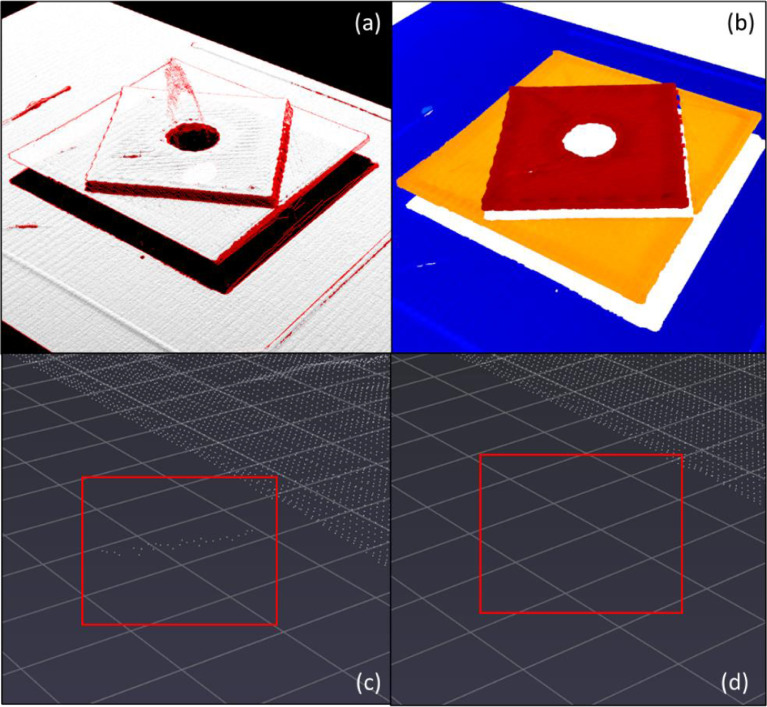
Effects of filtering for point cloud dataset (a) and (b) point cloud before and after applying statistical outlier removal algorithm, (c) and (d) point cloud before and after using region growing algorithm.

Due to the design of the laser scanner, the shadow effect can cause measurement noise in the raw data. When the scanner moves above areas of rapid change in height, these areas could be identified as outliers algorithmically according to the surrounding topographies. The statistical outlier removal algorithm and region growing algorithm in the point cloud library (PCL) [3] are customized and utilized to reduce the noise in the point cloud dataset. The effect of applying these algorithms is shown in Fig. 4 . Fig. 4 (a) highlights the noise point cloud and concentrates on the step area. In Fig. 4(b), to better display the point cloud after using a statistical outlier removal algorithm, the point cloud with different heights is rendered as different colors. It shows that the algorithm can effectively remove the noise between steps. Fig. 4(c) shows the noise point cloud as an island apart from the main body. The extra deposit plastic is as the print head travels through the open air and the laser scanner captures it. This phenomenon is commonly caused by incorrect retraction settings or high extruder temperatures. Since the study focuses on the upper place surface, the point cloud of extra material is treated as noise data and can be removed by using a region-growing algorithm.

### 3.4 Step 3: point cloud transformation

After removing the noise data, the point cloud should be transformed to the FDM printer coordinate to keep the scan results in the same spatial coordinate. Therefore, a calibration process is necessary to find the spatial relationship between the FDM printer and the laser scanner [4]. The spherical targets are used as calibration referencing markers. The calibration method is shown in Table 2. Four hemisphere targets are fabricated by the FDM printer. Since the fabrication coordinates of the hemisphere centers are known in the Gcode file, when the scan coordinates of the fabricated parts are obtained, the spatial relationship between FDM and laser scanner can be calculated and represented as 3D affine transformation matrix. To reduce the computational burden, transformation matrix R_1 is calculated to make the platform plane of point cloud parallel to the x-y plane of the FDM machine. Then transformation matrix R_2 is obtained by transferring one of the hemisphere centers to the design coordinate. Finally, the iterative closest point (ICP) algorithm is used to fine tune the alignment of all hemisphere centers and the design coordinates. The ICP algorithm is widely used to minimize the difference between two point clouds.

**Table 2:** Calibration algorithm for laser scanner.

Calibration for 3D printer and laser scanner system
**Input**: Point cloud of calibration target pt_scan, Vector of hemisphere centers in 3D printer coordinate v_target **Output**: Transformation matrix R 1. Denoise pt_scan, 2. Using RANSAC, segment platform plane plane_platform from pt_scan,get the normal vector of platform plane v_platform, 3. Obtain transformation matrix R_1 from v_platform to (0,0,1), 4. pt_1←R_1×pt_scan, 5. Segment hemispheres from pt_1, get the vector of hemisphere centers v_1, 6. Obtain transformation matrix R_2 from v_1 to v_target, 7. $R^{\prime }\leftarrow R\_2\;\times R\_1$ 8. Fine tune with Iterative Closest Point (ICP) algorithm $R\leftarrow R^{\prime }$

**Fig. 5:**
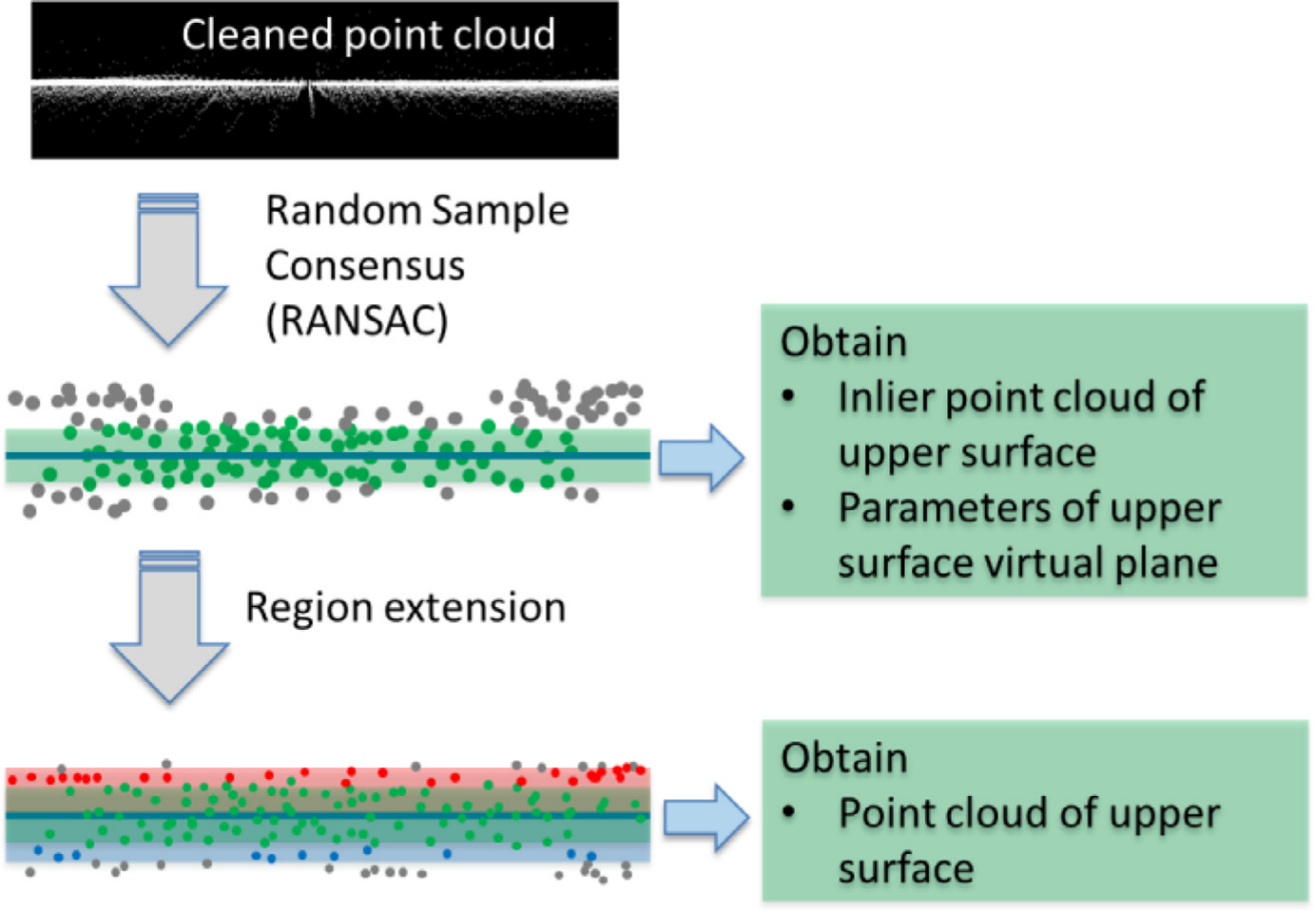
Upper surface segmentation.

Then the interested point cloud of the upper surface is segmented from the pre-processed point cloud. As shown in Fig. 5, the Random Sample Consensus (RANSAC) algorithm [5] is used to segment the planes of the point cloud. Using this algorithm, only the points between two parallel planes within distance threshold δ are considered as inliers. In this study, the δ is set as half of the layer thickness. The majority of the point cloud in the upper surface can be selected, shown as green points in Fig. 5. The plane segmented by RANSAC is identified as the upper surface virtual plane. Then the threshold δ is increased to the layer thickness value to include more point clouds, which are used as points of upper surface for the following processing.

**Fig. 6:**
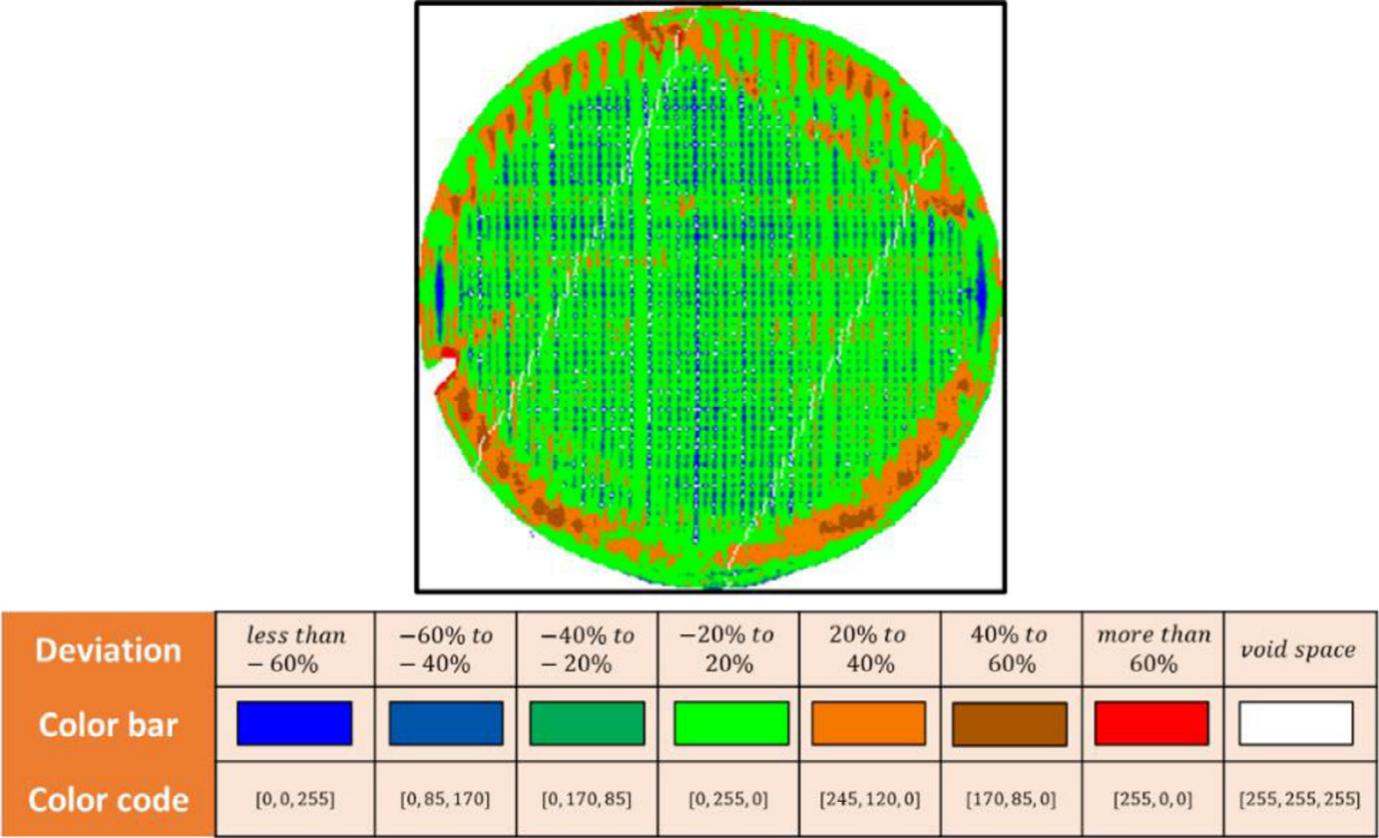
Color mapping rule for depth image, deviation values are determined by the ratio of the accumulated depth of pixel and layer thickness.

Finally, to generate a 2D depth image dataset shared in this paper, the point cloud of the upper surface needs to be projected on a 2D plane. The rasterization method is implemented [4]. Then the color of each pixel in the 2D depth image is determined by the ratio of the accumulated depth of pixel and layer thickness. For example, if the pixel is shown as green (0, 255, 0), it means that the accumulated depth of this pixel is between -20% to 20% of the layer thickness, which is 0.3mm in this study. The color mapping rule is shown in Fig. 6.

## 4 Database Structure

**Fig. 7:**
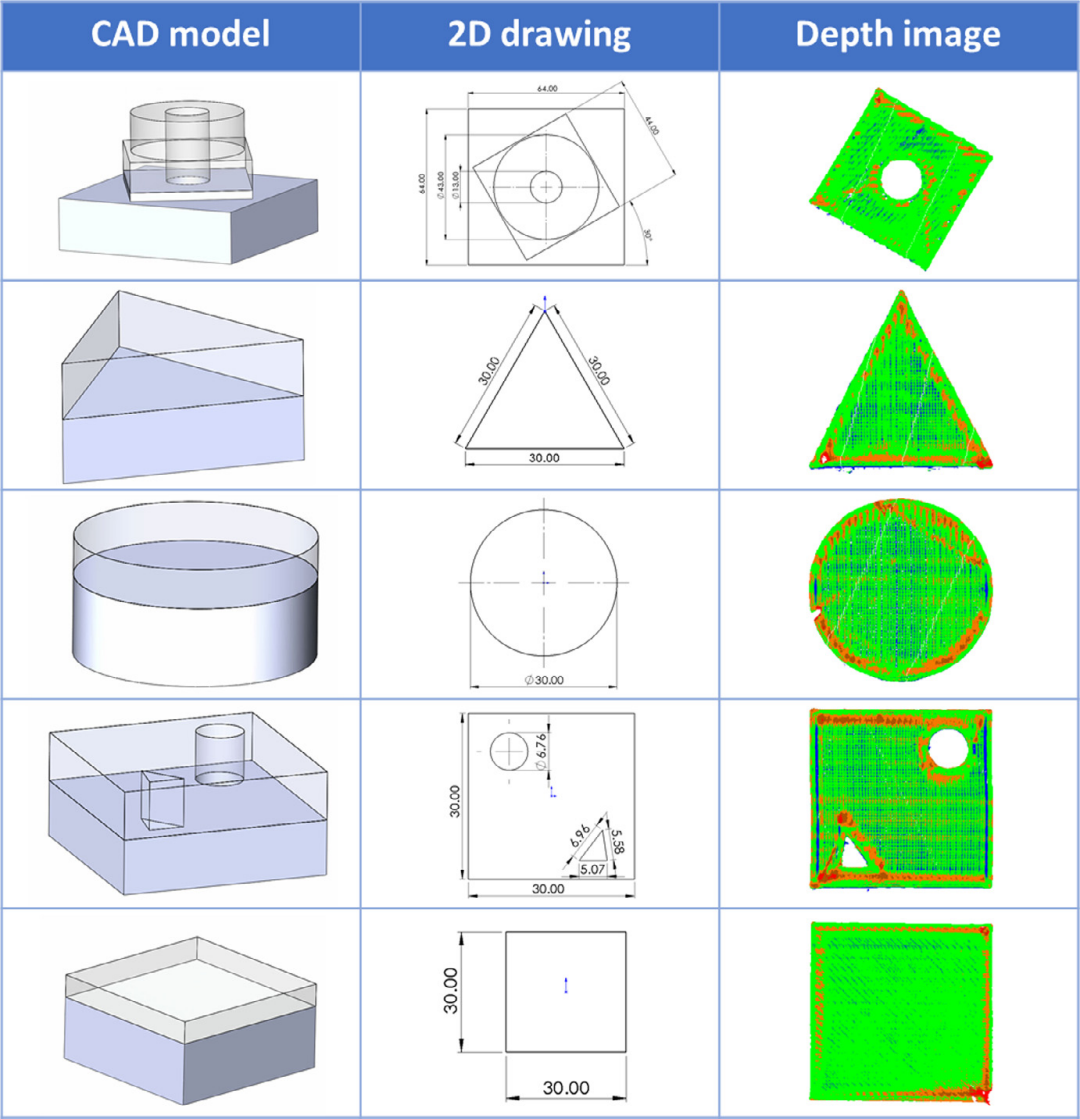
Artifacts fabricated by FDM machine.

The database is generated after the printing of multiple artifacts, shown in Fig. 7. During the printing of each artifact, with different settings, various qualities were observed. The process parameters are shown in Table 3. All the other parameters are kept constant. Later, each observation was studied and processed. In total, we gathered 434 scans, and by dividing each image into 10by10 segments, we generated 43400 labeled data images.

**Table 3:** FDM machine parameters.

FDM machine parameters	Value
Layer thickness	0.3 mm
Print speed	3500 mm/min
Infill density	100%
Feed rate	50% ∼ 150%
Extruder temperature	180°C ∼ 250°C

## 5 Labeling the Dataset

**Fig. 8:**
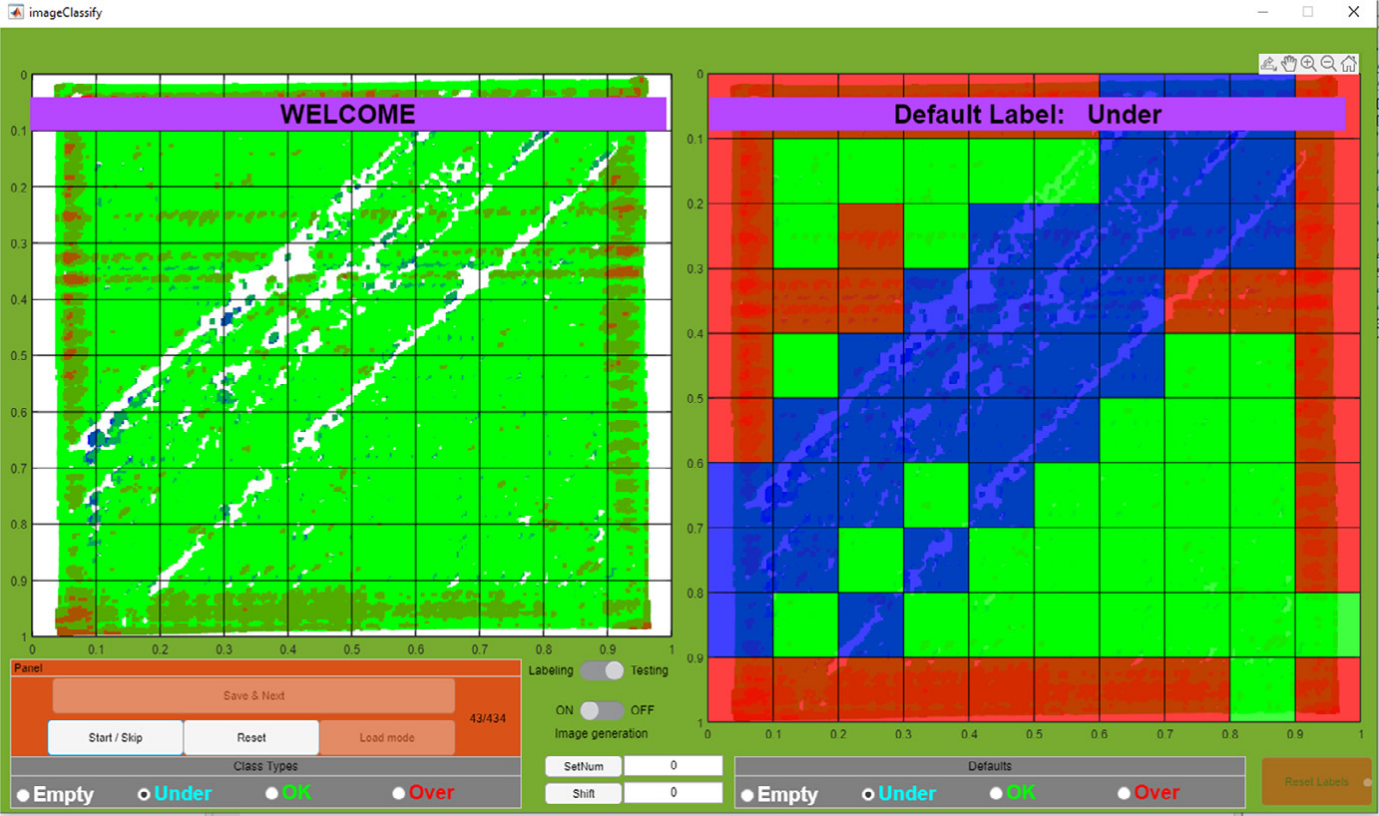
View of MATLAB Labeling application in review mode to visualize the labels recorded. Left, represents the part's heightmap. Right, the labeled map using solid colors for each label (Red for over extrusion, Blue for under extrusion, Green for normal and white for empty regions). White label is not demonstrated in this example.

Labeling a dataset in many cases requires a significant investment and effort of professionals to make sure the data is labeled correctly and in a repeatable nonbiased manner. In this dataset, to simplify the process, four categories for labels are selected: 1) Over Printing Situation, 2) Normally Printed Situation, 3) Under Printing Situation, 4) Empty Region. To better visualize the labeled data, solid color has been given to each grid based on its label respectively: Red, Green, Blue, white. Due to the complexity of the labeling task and the massive data, a User Interface based on MATLAB was developed to minimize the user error in labeling and store the labels by taking advantage of visual representation of data and labels. This Interface allows researchers to load their data, define the number of segments and labels needed, and ease the labeling process. Professionals can go over the previously labeled data with the review functionality to double-check and verify their accuracy visually. In Fig. 8, a sample of the labeling U.I. is shown in the Testing mode. This U.I. is also shared along with the data.

## Ethics Statements

The authors declare that the present work did not include experiments on human subjects and/or animals.

## CRediT Author Statement

**Jiaqi Lyu:** Conceptualization, Methodology, Software, Data curation, Writing- Original draft preparation, Visualization, Investigation; **Javid Akhavan:** Conceptualization, Methodology, Software, Data curation, Writing- Original draft preparation, Visualization, Investigation; **Souran Manoochehri:** Supervision

## Declaration of Competing Interest

The authors declare that they have no known competing financial interests or personal relationships that could have appeared to influence the work reported in this paper.

## Appendix Supplementary materials

Image, application 1
